# Sedentary behavior, brain-derived neurotrophic factor and brain structure in midlife: A longitudinal brain MRI sub-study of the coronary artery risk development in young adults study

**DOI:** 10.3389/frdem.2023.1110553

**Published:** 2023-03-13

**Authors:** Xuan Zhang, Osorio D. Meirelles, Zhiguang Li, Kristine Yaffe, R. Nick Bryan, Chengxuan Qiu, Lenore J. Launer

**Affiliations:** ^1^Laboratory of Epidemiology and Population Sciences Intramural Research Program, National Institute on Aging, Baltimore, MD, United States; ^2^Departments of Psychiatry and Behavioral Sciences, Neurology, and Epidemiology, University of California, San Francisco, San Francisco, CA, United States; ^3^Department of Radiology, University of Pennsylvania, Philadelphia, PA, United States; ^4^Aging Research Center and Center for Alzheimer's Research, Karolinska Institutet, Stockholm, Sweden

**Keywords:** sedentary time, brain volume, middle-aged, longitudinal, biomarkers

## Abstract

**Background:**

Brain-derived neurotrophic factor levels are higher in those who are physically active and lower in people with cognitive dysfunction. This study investigated whether brain-derived neurotrophic factor mediated or modified the association of sedentary time to MRI-estimated brain volumes in midlife.

**Methods:**

Baseline (*n* = 612) and five-year follow-up (*n* = 418) data were drawn from the multicenter Coronary Artery Risk Development in Young Adults Brain MRI sub-study, including Black and White participants (aged 50.3 years, 51.6% females, 38.6% Black). Sedentary time (hours per day) was categorized into quartiles with low ≤ 4.3 (reference) and high > 8.4. Outcomes of the study were total brain, white matter, gray matter, hippocampal volumes, and white matter fractional anisotropy at baseline and 5-year percent change from baseline. The study used general linear regression models to examine the mediation and moderation effects of brain-derived neurotrophic factor (natural log transformed) on the associations of sedentary time to brain outcomes. The authors adjusted the regression model for age, sex, race, intracranial volume, education, and vascular factors.

**Results:**

Cross-sectionally, baseline participants with the highest sedentary time had a lower total brain (−12.2 cc; 95%CI: −20.7, −3.7), gray matter (−7.8 cc; 95%CI: −14.3, −1.3), and hippocampal volume (−0.2 cc; 95%CI: −0.3, 0.0) compared with populations with the lowest sedentary time. The brain-derived neurotrophic factor levels did not mediate the associations between brain measures and sedentary time. Brain-derived neurotrophic factor was found to moderate associations of sedentary time to total brain and white matter volume such that the brain volume difference between high and low sedentary time decreased as brain-derived neurotrophic factor levels increased. Longitudinally, higher baseline brain-derived neurotrophic factor level was associated with less brain volume decline. The longitudinal associations did not differ by sedentary time, and brain-derived neurotrophic factor did not mediate or moderate the association of sedentary time to brain measure changes.

**Conclusions:**

Higher brain-derived neurotrophic factor levels may buffer the negative effects of sedentary time on the brain.

## Introduction

Several studies suggest that high levels [e.g., >5 h per day or 3.0 metabolic equivalents (MET)] of physical activity (PA) are associated with healthier brain structures and functions in middle-aged (Chang et al., 2010; Hoang et al., 2016; Young et al., 2016) and older persons (Burzynska et al., 2014; Barha et al., 2019). The converse, sedentary behavior, such as television viewing or sitting (e.g., >8 h per day or energy expenditure of 1.5 MET or less), has been shown to be associated with lower brain volumes and poorer cognitive function than an active lifestyle (Launer et al., 2015; Hoang et al., 2016; Young et al., 2016).

Although an association between brain health and levels of PA has been reported, there are few studies identifying intermediaries (mediators) of this association, or factors that may change the direction of the association (moderators) (Kramer, 2021). Brain-Derived Neurotrophic Factor (BDNF) (Bekinschtein et al., 2007; Raz et al., 2009; Brown et al., 2014; Shimada et al., 2014; Weinstein et al., 2014) is a neurotrophic and neuroprotective protein that maintains brain health (Shimada et al., 2014; Weinstein et al., 2014; Li et al., 2020). Is BDNF a mediator or moderator? High levels of PA are associated with relatively higher BDNF plasma levels, which have a positive effect on brain structure and function (Vaynman et al., 2004; Raz et al., 2009; Brown et al., 2014). Likewise experimental studies have shown that blocking BDNF signaling pathways could inhibit the benefits of high activity levels on cognitive and hippocampal function in the memory domain (Vaynman et al., 2004; Bekinschtein et al., 2007; Korol et al., 2013). Additional evidence from genetic studies suggests levels of BDNF secreted from virus-infected neurons are regulated by the BDNF Val66 Met polymorphism and that higher levels of PA were associated with larger regional brain volumes and better cognitive function in Val/Val homozygotes than Met carriers and there were smaller regional volumes in Met carriers than Val/Val homozygotes (Egan et al., 2003; Brown et al., 2014; Takeuchi et al., 2020). Sedentary behavior connotates low levels of activity. There is variability of sedentary time (ST) in the population, and the amount of ST spent by an individual is associated with chronic disease independent of moderate to vigorous PA (Owen et al., 2010). For example, sitting for more than 10 h per day vs. < 5 h per day was associated with an increased risk of CVD among middle-aged women in the Women's Health Initiative population (Young et al., 2016).

**Figure 1:**
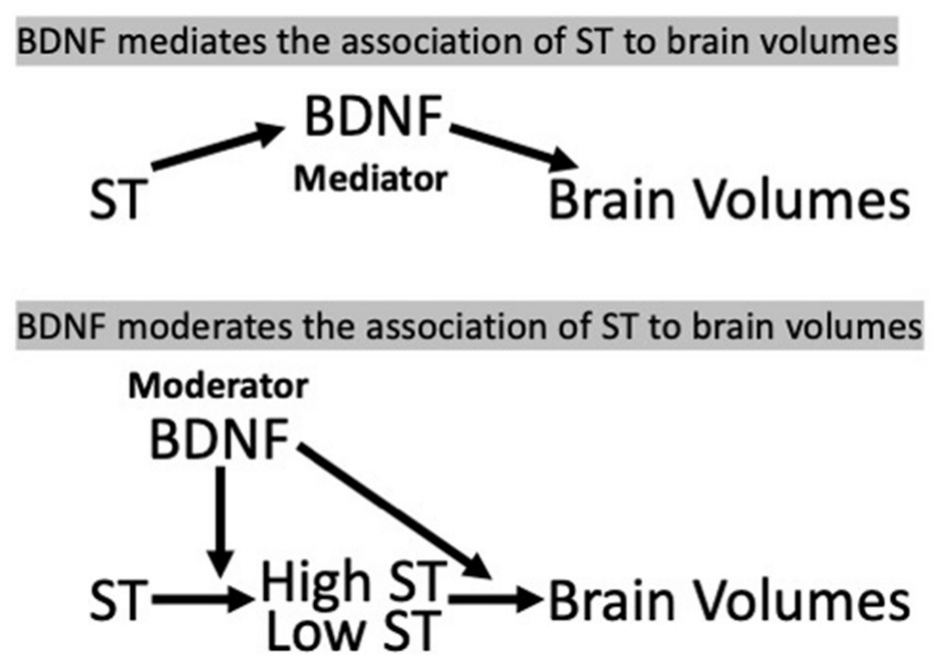
Hypothesis 1 (mediation) and Hypothesis 2 (moderation/interaction) model, CARDIA Brain-MRI sub-study. (a) BDNF, brain-derived neurotrophic factor; ST, sedentary time.

Based on our previous finding of a relation between time spent in sedentary behaviors and total brain volume (TBV) (Launer et al., 2015), we investigated whether BDNF levels mediated (in the pathway of ST and brain measures) or moderated (interaction with ST) the ST—brain structure associations (Figure 1) in a cross-sectional and longitudinal analysis.

## Materials and methods

### Study population

This study is based on Coronary Artery Risk Development in Young Adults study (CARDIA), a community-based longitudinal study of Black and White men and women aged 18–30 years at baseline, who had been followed up for 30 years by the time of this study and has previously been described in Friedman et al. (1988). The CARDIA brain MRI sub-study was started in the 25th year (Y25) of follow-up and repeated in the 30th year (Y30) of follow-up (Launer et al., 2015). Briefly, the sub-study aimed to characterize the morphology, pathology, physiology, and function of the brain using MRI technology. Participants were enrolled at the Y25 exam with the aim to achieve a balance of ethnicity/race and sex from three of the four CARDIA field centers: Birmingham, AL, Minneapolis, MN, and Oakland, CA. Each center recruited to a target of approximately 200 individuals. Exclusion criteria were contra-indication to MRI or a too large body size for the MRI tube bore. Ethics approval was given by the institutional review boards from each field center and coordinating center (University of Alabama Birmingham, University of Minnesota, and Kaiser Permanente Northern California), the MRI Reading Center (University of Pennsylvania) and the NIH Office of Human Subjects Research Protection for the Intramural Research Program, National Institute on Aging. All participants provided written informed consent at each CARDIA exam, with a separate written consent for participation in the brain MRI sub-study.

### Assessment of brain structure

Brain MRI acquisition and processing information have been previously reported (Launer et al., 2015). Each CARDIA clinic site acquired brain MRI on 3-T MR scanners and transferred MRI data to a central archive, the MRI Reading Center, located at the University of Pennsylvania. We included the following in our analysis: volumetric (cubic centimeters, cc) estimates of total brain (TBV), gray matter (GMV), white matter (WMV), and the hippocampus (HV). We also explored WM tissue integrity measured by fractional anisotropy (WMFA) from diffusion tensor imaging scans (Haight et al., 2018).

### Assessment of main variables of interest

ST was estimated by average hours per day from self-reported time spent (0, 15 mins or less, 30 mins, 1–5 h respectively, and 6 h or more) in the following activities: sitting while watching television, using the computer, doing non-computer paperwork, listening to music, reading, or doing arts, using the phone, and riding transportation. The details of the questionnaire can be found on the CARDIA website (https://www.cardia.dopm.uab.edu/images/more/pdf/Year25/CARDIA/Form91.pdf). We calculated the ST per week by adding up the reported hours in specific activities weighted by weekdays (5/7) and weekends (2/7). Plasma BDNF levels (picograms/ml, pg/ml) from specimens collected at the Y25 exam were assayed using the Quantikine^®^ Human Free BDNF Immunoassay. Free BDNF in plasma, which was not bound to other proteins, receptors, or other factors, was measured by a 3.5-h solid phase ELISA that possesses a detectable range of 125–40,000 pg/ml at a 1:10 dilution.

### Assessment of covariates

Based on prior studies, we included in our analysis demographic factors (i.e., age, sex, and race) (Raz et al., 2009; Liu et al., 2016; Brown et al., 2020), and total intracranial volume (ICV), as a measure of head size (Driscoll et al., 2012).

We also included in our models, cardiovascular risk factors, which have been shown to be associated with plasma BDNF levels, and with brain volumes. High levels of vascular risk factors are generally associated with brain atrophy (Launer et al., 2015; Cox et al., 2019), while the relationship between vascular factors and BDNF is inconsistent and may depend on age and sex (Pikula et al., 2013; Jamal et al., 2015).

Other factors included in the models were: Education levels (Chan et al., 2018); BMI (kg/m^2^) (Ho et al., 2011); tobacco use (ever/never) (Pikula et al., 2013); presence of diabetes (based on fasting glucose, glucose tolerance, hemoglobin A1c, and medications) (American Diabetes Association, 2011), hypertension (based on: ≥ 140/≥90 mmHg or on hypertensive medications) (Chobanian et al., 2003), dyslipidemia (based on: triglycerides ≥ 150 mg/dl, HDL < 35 mg/dl if males; Triglycerides ≥ 150 mg/dl, HDL < 45 mg/dl if females) (Gottesman et al., 2017), and depression symptomology measured by the Center for Epidemiologic Studies Depression Scale (Beekman et al., 1997).

### Analytical sample

At the Y25 examination, 719 men and women participated in the Brain MRI Sub-study, among whom 625 had plasma BDNF data (Supplementary Figure 1). After excluding 13 persons with missing values of ST data, there were 612 with complete data for these analyses. Secondary longitudinal analyses included 418 (68.3%) participants who attended both the Y25 and Y30 MRI exams and had complete data. There were 194 (31.7%) participants who did not return for the second MRI (Supplementary Table 1). In general, the dropouts had a higher ST and BMI, and more often had diabetes, hypertension, dyslipidemia, hypertension, and smoked. However, both the baseline BDNF and brain measures were similar among the dropouts and attenders.

### Statistical analyses

All brain measures were normally distributed and coded as continuous variables. ST distribution was right skewed. To compare high ST with low ST, ST was categorized into quartiles with the lower 25%ile at ≤ 4.3 h per day as the reference group. We used natural log transformation for the distribution of plasma BDNF levels to normalize the right-skew. Age was a continuous variable. Binary variables included sex (female and male) and race (White and Black) with males and Blacks as the reference groups, respectively.

For descriptive analysis, we used ANCOVA and logistic regression to compare continuous and categorical characteristics at the Y25 exam across the ST quartiles, respectively. For Model 1, we included age, sex, race, and ICV as covariates. Model 2 (supplement) additionally included vascular risk factors, as described above. To test for mediation effects of BDNF (Hypothesis 1), we used general linear regression to examine the associations of ST to MRI outcomes, with and without BDNF in the model. We tested whether levels of BDNF moderated the association of ST to brain measures by entering an interaction term (Hypothesis 2), BDNF by ST. Multicollinearity was checked between all predictor variates with all correlation coefficients < 0.6.

To verify and to expand this basic statistical approach, we also conducted a formal mediation and moderation analyses using the PROCESS model (Hayes, 2018). In these analyses, the direct and indirect (mediation) effects of ST on brain outcomes were estimated, with a test of the significance of BDNF as a mediator. Similarly, the PROCESS analysis tests for moderation effects (Hayes, 2018) by estimating coefficients of the model where brain measures are dependent variables, ST an independent variable, BDNF as the moderating variable, adjusting for covariates. The PROCESS analysis gives values of brain measures by ST quartiles at the 16, 50, and 84%ile of logBDNF.

For the longitudinal analysis, we used the same approach as in the main analysis. Five-year brain measure change was expressed as the percent change in brain measures from Y25 to Y30 exam (Jack et al., 2004).

We conducted all analyses using SAS 9.4^®^ (SAS Institute, Inc., Cary, NC). Significance was based on 95% CI not including 0.

## Results

### Characteristics of study participants at the year 25

At the Y25 exam, participants were on average 50.3 years old, 51.6% were female, and 38.6% were Black (Table 1). Black participants tended to have higher ST. Vascular risk factors varied differed across ST quartiles. People with higher ST quartiles had higher BMI, and more often had diabetes, hypertension, dyslipidemia, and smoked compared with people in the lowest ST quartile (Table 1, Supplementary Table 3). Smoking (β = 0.23, 95% CI: 0.07, 0.40) and hypertension (β = 0.21, 95% CI: 0.04, 0.39) were significantly associated with one-unit higher logBDNF levels adjusting for age, sex, and race, respectively, compared with never-smoking and non-hypertensive (Table 2).

**Table 1:** Characteristics of participants at the year 25, CARDIA brain-MRI sub-study.

**Characteristics**	**Total**	**Sedentary time (hours per day), quartiles**			
		≤ **4.3**	**4.3–5.9**	**5.9–8.4**	>**8.4**
***n***	**612**	**152**	**154**	**152**	**154**
Age, mean (SD), years	50.3 ± 3.5	50.6 ± 3.5	50.4 ± 3.5	50.5 ± 3.5	49.8 ± 3.6
**Sex no. (%)**					
Male	296 (48.4)	69 (45.4)	74 (48.1)	73 (48.0)	80 (52.0)
Female	316 (51.6)	83 (54.6)	80 (52.0)	79 (52.0)	74 (48.1)
**Race no. (%)**					
Black	236 (38.6)	35 (23.0)	43 (27.9)	60 (39.5)	98 (63.6)
White	376 (61.4)	117 (77.0)	111 (72.1)	92 (60.5)	56 (36.4)
**Education no. (%)**					
High school	131 (21.5)	22 (14.5)	30 (19.5)	29 (19.2)	50 (32.7)
College	356 (58.4)	92 (60.5)	84 (54.6)	94 (62.3)	86 (56.2)
Graduate	123 (20.2)	38 (25.0)	40 (26.0)	28 (18.5)	17 (11.1)
^*^BMI, mean (SD), kg/m^2^	28.7 ± 5.7	26.6 ± 5.2	28.0 ± 5.0	30.1 ± 6.3	30.2 ± 5.4
^*^Diabetes no. (%)	67 (11.0)	7 (4.6)	13 (8.4)	22 (14.5)	25 (16.2)
^*^Dyslipidemia no. (%)	235 (38.5)	42 (27.6)	61 (39.6)	65 (43.1)	67 (43.5)
^*^Hypertension no. (%)	198 (32.4)	29 (19.1)	33 (21.4)	64 (42.1)	72 (46.8)
^*^Smoking no. (%)	240 (39.6)	48 (32.0)	55 (35.7)	65 (43.1)	72 (47.7)
Depression score, mean (SD)	8.6 ± 7.0	7.6 ± 6.7	8.5 ± 6.3	8.6 ± 7.5	9.6 ± 7.4
BDNF plasma, mean (SD), pg/ml	3,090.0 ± 3,045.7	2,934.0 ± 2,898.3	2,859.9 ± 2,333.2	3,113.5 ± 3,179.0	3,450.9 ± 3,623.4
**Brain measures, % of ICV (SD)**					
TBV	85.1 ± 2.8	85.2 ± 2.3	85.1 ± 0.3	85.3 ± 2.9	84.8 ± 3.3
GMV	46.8 ± 2.2	46.8 ± 1.9	46.8 ± 2.2	46.9 ± 2.3	46.5 ± 2.7
WMV	38.3 ± 1.6	38.4 ± 1.5	38.3 ± 1.5	38.3 ± 1.7	38.3 ± 1.7
HV	0.6 ± 0.1	0.6 ± 0.0	0.6 ± 0.1	0.6 ± 0.1	0.6 ± 0.1
WMFA, mean^*^10^−2^ (SD)	31.1 ± 1.8	31.2 ± 1.7	31.4 ± 1.8	31.2 ± 1.6	30.7 ± 2.1
**% change from year 25, mean (SD)**					
TBV^*^	0.4 ± 1.6	0.5 ± 1.8	0.4 ± 1.5	0.5 ± 1.5	0.3 ± 1.9
GMV	−0.3 ± 2.3	−0.2 ± 2.3	−0.4 ± 2.4	−0.2 ± 2.1	−0.6 ± 2.5
WMV	1.3 ± 1.9	1.3 ± 1.9	1.4 ± 1.7	1.4 ± 1.9	1.4 ± 2.2
HV	0.3 ± 2.4	0.5 ± 2.8	0.4 ± 2.1	0.4 ± 2.3	−0.0 ± 2.5
WMFA	−2.8 ± 3.1	−2.9 ± 3.5	−2.3 ± 2.3	−3.0 ± 3.1	−3.0 ± 3.5

BDNF, brain-derived neurotrophic factor; BMI, body mass index; ICV, intracranial volume; TBV, total brain volume; GMV, gray matter volume; WMV, white matter volume; HV, hippocampal volume; WMFA, white matter fractional anisotropy. Missingness: Education n = 2; Dyslipidemia n = 2; Depression score n = 3.

^*^P-value < 0.05, adjusted for age, sex, race, and ICV.

**Table 2:** Associations between logBDNF and 25-year exam risk factors, CARDIA brain-MRI sub-study.

**Risk factor**	**β estimate**	**95%CI**
Education	−0.10	−0.24, 0.03
BMI	0.01	−0.01, 0.02
Diabetes	0.04	−0.23, 0.31
Smoking	0.23	0.07, 0.40
Hypertension	0.21	0.04, 0.39
Dyslipidemia	0.13	−0.03, 0.30
Depression	−0.01	−0.02, 0.01

BDNF, brain-derived neurotrophic factor; BMI, body mass index.

β estimate was the difference of logBDNF per-unit change for yes vs. no risk factors, adjusted for age, race, and sex.

### Hypothesis 1: Cross-sectional mediation effects of BDNF

Adjusting for age, sex, race, and ICV, compared with those in the lowest ST quartile ( ≤ 4.3 h per day), people in the highest ST quartile (>8.4 h per day) had a significantly smaller brain volumes: TBV of −12.2 cc (95%CI: −20.73, −3.69), GMV of −7.8 cc (95%CI: −14.31, −1.28), and HV of −0.2 cc (95%CI: −0.33, −0.03) (Table 3). There were no significant differences in WMV or WMFA across different ST quartiles. Neither ST quartiles nor any of the brain measures were significantly associated with BDNF. Controlling for BDNF, or other covariates, the indirect effects between ST and brain measures were similar to the direct effects (i.e., without BDNF adjustment) suggesting BDNF did not mediate the association between ST and brain structure.

**Table 3:** Mediation and moderation effects of plasma BDNF levels on sedentary time (ST) and brain measures, model 1, CARDIA brain-MRI sub-study.

**Models**	**Cross-sectional**	**Longitudinal**
	β **coefficient (95%CI)**	β **coefficient (95%CI)**
**Hypothesis 1: Mediation**		
**ST (highest vs. lowest quartile)**→**MRI**		
TBV	−12.2 (−20.73, −3.69)	−0.2 (−0.65, 0.32)
GMV	−7.8 (−14.31, −1.28)	−0.4 (−1.09, 0.28)
WMV	−4.4 (−9.43, 0.60)	0.2 (−0.40, 0.70)
HV	−0.2 (−0.33, −0.03)	−0.5 (−1.25, 0.17)
WMFA	−0.3 (−0.74, 0.08)	−0.1 (−1.14, 1.01)
**ST (highest vs. lowest quartile)** ** → logBDNF**	0.2 (−0.06, 0.42)	N/A
**logBDNF**→**MRI**		
TBV	−0.9 (−3.74, 2.01)	0.3 (0.14, 0.45)
GMV	−0.8 (−2.98, 1.41)	0.3 (0.12, 0.56)
WMV	−0.1 (−1.76, 1.61)	0.2 (0.06, 0.41)
HV	0.0 (−0.03, 0.07)	0.5 (0.29, 0.74)
WMFA	−0.1 (−0.23, 0.05)	−0.5 (−0.87, −0.18)
**ST (highest vs. lowest quartile)**→**logBDNF**→**MRI**		
TBV	−12.1 (−20.65, −3.57)	−0.2 (−0.69, 0.26)
GMV	−7.7 (−14.23, −1.17)	−0.5 (−1.14, 0.21)
WMV	−4.4 (−9.45, 0.62)	0.1 (−0.44, 0.66)
HV	−0.2 (−0.33, −0.03)	−0.6 (−1.33, 0.06)
WMFA	−0.3 (−0.73, 0.10)	0.0 (−1.04, 1.09)
**Hypothesis 2: Moderation**		
**ST (highest vs. lowest quartile) and logBDNF moderation**		
TBV	8.4 (0.28, 16.54)	−0.4 (−0.82, 0.06)
GMV	2.6 (−3.69, 8.79)	−0.5 (−1.09, 0.15)
WMV	5.9 (1.08, 10.64)	−0.3 (−0.79, 0.23)
HV	0.1 (−0.07, 0.21)	−0.6 (−1.19, 0.08)
WMFA	0.2 (−0.15, 0.63)	0.2 (−0.75, 1.24)

BDNF, brain-derived neurotrophic factor; ST, sedentary time; TBV, total brain volume; GMV, gray matter volume; WMV, white matter volume; HV, hippocampal volume; WMFA, white matter fractional anisotropy.

Results were based on Model 1, adjusted for age, sex, race, and intracranial volume (ICV). For Hypothesis 1, β coefficients were for ST on logBDNF, MRI (cross-sectional) or change of MRI (longitudinal). For Hypothesis 2, β coefficients were for interaction.

### Hypothesis 2: Cross-sectional moderation (interaction) analysis

**Figure 2:**
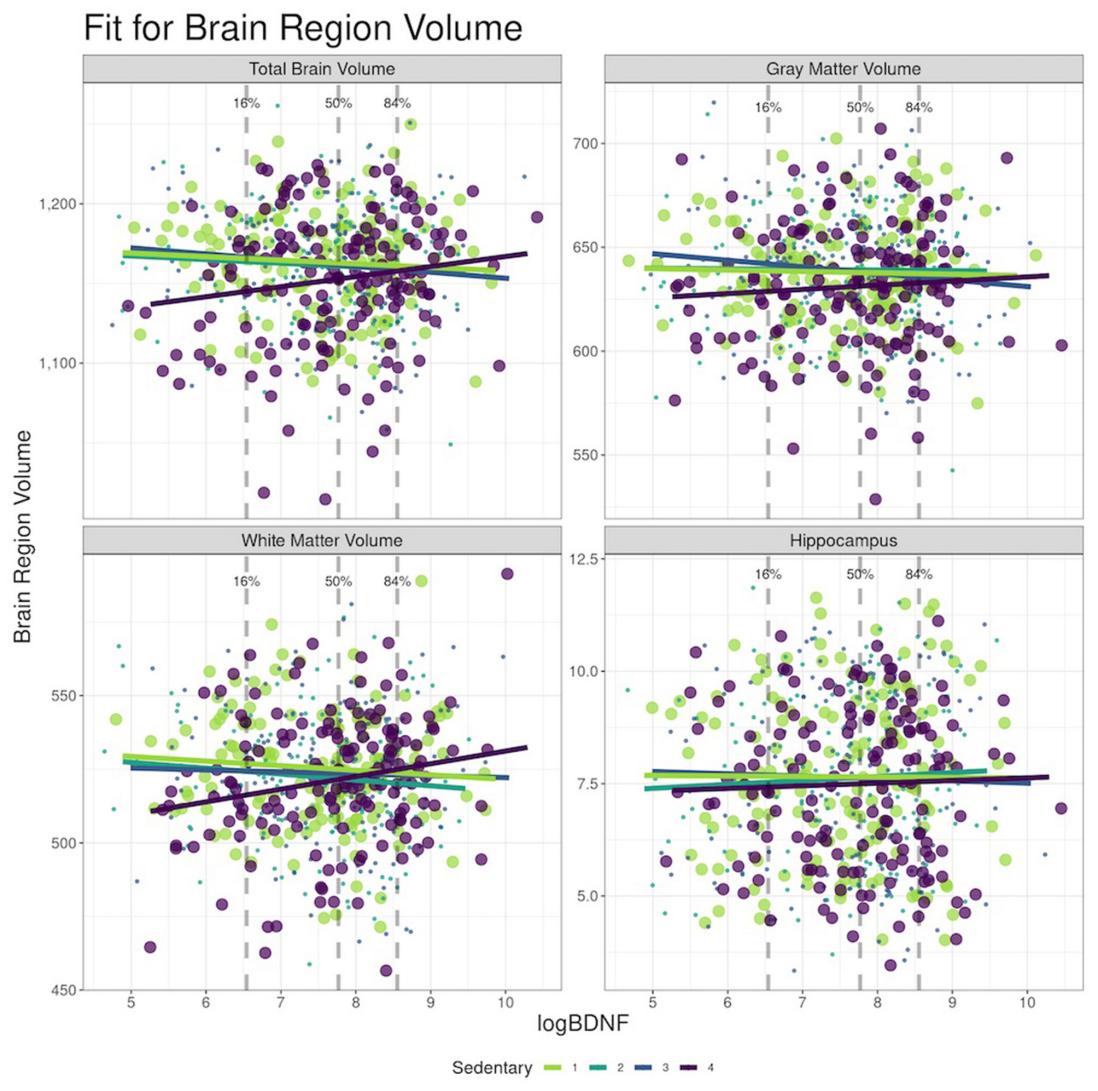
Moderation effects of BDNF on sedentary time (ST) to brain MRI at the Year 25, CARDIA Brain-MRI sub-study. (a) BDNF, brain-derived neurotrophic factor. (b) Models adjusted for age, sex, race, and intracranial volume (ICV).

In Model 1 (Table 3, Figure 2), plasma BDNF significantly moderated the difference in TBV (95% CI_interaction_: 0.28, 16.54) and WMV (95% CI_interaction_: 1.08, 10.64) between the highest and lowest ST quartile. In those in the highest quartile of ST, higher BDNF levels were associated with higher TBV and WMV. Therefore, the difference in TBV and WMV between the high and low quartiles of ST was reduced with higher BDNF levels. Controlling for covariates, the difference in TBV between the high and low ST quartiles at the 16th percentile of logBDNF was −21.4 cc (95% CI: −33.60, 9.21), −11.0 cc (95% CI: −19.65, −2.41) at the 50th logBDNF percentile, and a non-significant difference of −4.4 cc (95% CI: −15.89, 7.13) at the 84th percentile. For WMV, the difference between the high and low quartiles was −10.8 cc (95% CI: −18.01, −3.66), −3.6 cc (95% CI: −8.67, 1.47) and 1.0 cc (95% CI: −5.74, 7.80) for the 16th, 50th and 84th logBDNF percentiles respectively. In Model 2, after additionally adjusting for vascular risk factors, plasma BDNF significantly moderated the difference in WMV (95% CI_interaction_: 1.14, 11.49) but not the TBV (95% CI_interaction_: −0.13, 17.11) between the high and low ST quartiles (Supplementary Table 1).

### Hypotheses 1 and 2 longitudinal analyses

Longitudinally, there was no significant difference in percent change in brain measures by ST quartiles adjusting for age, sex, race, and ICV (Table 3). However, the amount of change in brain volumes depended on the BDNF level. For example, with every unit increase in logBDNF, there was a 0.3% (95% CI: 0.14%, 0.45%) less decline in TBV. Table 3 also shows similar associations in GMV, WMV, and HV. There was no evidence that BDNF mediated or moderated the association of ST to change in brain measures.

## Discussion

Based on cross-sectional and longitudinal analyses, we investigated whether level of plasma BDNF influenced the association between ST and brain structure in a community-dwelling, middle-aged cohort of Black and White participants. Plasma BDNF levels modified the association between ST and brain measures. We found the lower the ST, the lower the brain volumes, but the differences between brain volumes in high vs. low ST were smaller as logBDNF increased in TBV and WMV (Figure 2). Our longitudinal analyses showed no association of ST to change in brain volumes but did show less decline in brain volume with higher BDNF levels. We did not find significant mediation or moderation effects in WM integrity in our study population.

### The activity-BDNF-brain volume associations

BDNF plays a neurotrophic role for neuronal survival and brain plasticity for cognitive function (Weinstein et al., 2014). Experimental and human studies have linked plasma BDNF levels to brain structure and function and have shown exercise increased BDNF levels and brain health. However, prior studies on mediation or moderation of BDNF plasma levels on the PA—brain function are limited, and the findings are inconsistent (Egan et al., 2003; Vaynman et al., 2004; Raz et al., 2009; Brown et al., 2014). A recent review of randomized controlled trials by Gasquoine suggested limited benefits of PA for memory performance through BDNF regulation, suggesting pathways outside the central nervous system regarding the beneficial effects of PA for cognitive function (Gasquoine, 2018).

In general, mechanisms that regulate plasma BDNF levels are not clear. Several studies have shown plasma BDNF levels are under genetic regulation of the BDNF Val66Met polymorphism where Met allele carriers have lower plasma BDNF levels than Val/Val homozygotes (Takeuchi et al., 2020). There are also differences in brain structure or function associated with Met and Val carriership (Brown et al., 2014; Weinstein et al., 2014). Other studies have suggested plasma BDNF levels are influenced by lifestyle factors either directly or indirectly by the BDNF Val66Met polymorphism (Egan et al., 2003). These factors include cognitive stimulation (Adcock et al., 2020), diet (Sánchez-Villegas et al., 2011; Yamada-Goto et al., 2012), stress (Yamada-Goto et al., 2012), sleep (Schmitt et al., 2016), and smoking (Xia et al., 2019). In our study, tobacco use, and hypertension were related to significantly higher plasma BDNF levels (Table 2). This finding is consistent with studies in mid- to late-life populations and in animal models (Kenny et al., 2000; Golden et al., 2010; Amoureux et al., 2012; Weinstein et al., 2014). BDNF activity, the amount of BDNF expressed by the regulating genes, was positively correlated with high blood pressure and negatively associated with smoking (Golden et al., 2010; Weinstein et al., 2014). In animal studies, increased aortic BDNF expression occurs before blood pressure increases (Amoureux et al., 2012). Regional brain BDNF expression increased after chronic nicotine treatment in rats through the BDNF-TrkB pathway (Kenny et al., 2000).

We also found no association of ST to BDNF, suggesting measures of low levels of activity do not capture low levels of exposure to BDNF-associated factors that are specifically stimulated by exercise. These processes may be independent of those that lie along the sedentary—active continuum or are dependent on other lifestyle or disease-related associated processes.

### BDNF and the brain

Our findings from the longitudinal analyses suggest that relatively higher BDNF levels are associated with less 5-year decline in TBV, GMV, and HV. Although many studies have focused on maintaining BDNF levels for the benefits of GM structures such as the hippocampus (Erickson et al., 2010; Maioli et al., 2012), there is also evidence that BDNF may play a role in maintaining WM health through expression by glial cells (Sato et al., 2009; Giorgio et al., 2010; Miyamoto et al., 2015). Studies have reported relatively higher BDNF levels were associated with slower change in WMV with age (Driscoll et al., 2012). However, relatively higher BDNF plasma levels are associated with more 5-year decline in WM microstructure measured by WMFA in our study. This relationship could be explained by the upregulation of BDNF levels through oligodendrogenesis by WM glial cells (e.g., astrocytes) to protect and regenerate damaged WM fibers (Sato et al., 2009; Giorgio et al., 2010; Miyamoto et al., 2015).

### Strengths of the study

This study has several strengths. As mentioned in previous publications, the CARDIA study provides data on a large community-based middle-aged cohort that includes Black and White participants. Data on this midlife age group provide important information of early changes in the brain, which may contribute to late-age prediction of cognitive impairment. Further, there are few data on the associations among ST, BDNF and brain volume in a such a cohort, which reflects the wider community. Additionally, we could examine these associations both cross-sectionally and longitudinally. We were also able to systematically study and identify other lifestyle factors that may influence levels of BDNF and should be accounted for in future studies.

### Limitations of our study

Although the people who returned for a second MRI had fewer cardiovascular risk factors, there was no difference in BDNF and brain measures compared to those who did not return. Compared to studies with objective measures of PA, our self-reported ST may be over or underestimated to an unknown degree particularly if there are systematic differences in reporting by a factor of importance to this study, such as smoking or hypertension (Wanner et al., 2017). Furthermore, ST not only includes relatively inactive sitting, but it also includes activities such as computer work, socializing using the phone, or artwork. There are mixed associations between different sedentary activities and the brain, including both positive impact of mentally stimulating activities (e.g., crafts, puzzles, video games, etc.) (Wanders et al., 2021) and a negative impact from TV viewing (Hoang et al., 2016). More complete measures of sedentary behavior which could differentiate time and activities on sitting at work and during leisure time are needed to further explore the effect of cognitive stimulation on BDNF levels and its association with brain outcomes.

Lastly, we were not able to examine the role of the BDNF polymorphism in our study. Additionally, the analysis assumes the BDNF plasma levels reflect its activity in the brain, which is a weakness in all studies of plasma biomarkers of brain health. In humans, peripheral BDNF is often used as a proxy since central BDNF is difficult to measure. In animal studies, the peripheral and central BDNF are associated by BDNF crossing the blood-brain barrier in both directions (Gejl et al., 2019). However, as with all blood-based biomarkers of central processes, there is always a gap in our understanding of how close the blood-based molecules reflect central activity.

## Conclusions

Together with other experimental and clinical studies, this study suggests that the negative association of ST with TBV and WMV was reduced in persons with relatively higher plasma BDNF levels in midlife.

## Data availability statement

The original contributions presented in the study are included in the article/Supplementary material, further inquiries can be directed to the corresponding author.

## Author contributions

XZ had full access to all of the data in this study and takes responsibility for the integrity of the data and the accuracy of the data analysis. Concept, design, and drafting of the manuscript: XZ, CQ, and LL. Acquisition, analysis, or interpretation of data: XZ, ZL, CQ, and LL. Critical revision of the manuscript for important intellectual content: XZ, OM, ZL, KY, RB, CQ, and LL. Statistical analysis: XZ, OM, ZL, CQ, and LL. Technical support: ZL. All authors contributed to the article and approved the submitted version.
