# Supplementary material for: Sedentary behavior, brain-derived neurotrophic factor and brain structure in midlife: A longitudinal brain MRI sub-study of the coronary artery risk development in young adults study
Source: Front Dement. 2023 Mar 13;2:1110553. doi: 10.3389/frdem.2023.1110553 (PMC11285629; doi:10.3389/frdem.2023.1110553)
Supplement: Supplementary file 1 [file Data_Sheet_1.docx]

Supplementary Material

Sedentary Behavior, Brain-Derived Neurotrophic Factor and Brain Structure in Midlife:

A Longitudinal Brain MRI Sub-study of the Coronary Artery Risk Development in Young Adults Study

Xuan Zhang, Osorio D Meirelles, Zhiguang Li, Kristine Yaffe, R Nick Bryan, Chengxuan Qiu, Lenore J Launer*

*** Correspondence:** Lenore J Launer: [launerl@nia.nih.gov](mailto:launerl@nia.nih.gov)

**Figure 1_Supp. Consort flowchart of participants enrollment, CARDIA Brain-MRI sub-study**

**Excluded (n=107)**

- **No plasma BDNF (n=94)**
- **No sedentary behavior data (n=13)**

**Brain MRI Sub-study at the Year 25 (n=719)**

**Excluded (n=194)**

- **No brain volumes data at Year 30**

**Included at Year 25 (n=612)**

**Included for brain volume analysis at Year 30 (n=418)**

**Figure 2_Supp. Moderation effects of BDNF on sedentary time (ST) to DTI at the Year 25, CARDIA Brain-MRI sub-study**

See attached figure.

Note.

1. BDNF=brain-derived neurotrophic factor, DTI=diffusion tensor imaging, WMFA=white matter fractional anisotropy.
2. Models adjusted for age, sex, race, and intracranial volume (ICV).

**Figure 3_Supp. Moderation effects of BDNF on TV viewing to Brain MRI at the Year 25, CARDIA Brain-MRI sub-study**

See attached figure.

Note.

1. BDNF=brain-derived neurotrophic factor.
2. Models adjusted for age, sex, race, and intracranial volume (ICV).

**Table 1_Supp. Mediation and moderation Effects of plasma BDNF Levels on sedentary time (ST) and brain measures, Model 2, CARDIA Brain-MRI sub-study**

|  | **Cross-sectional** | **Longitudinal** |
| --- | --- | --- |
| **Models** | **β coefficient (95%CI)** | **β coefficient (95%CI)** |
| **Hypothesis 1: Mediation** |  |  |
| **ST (highest vs. lowest quartile) → MRI** |  |  |
| TBV | -9.9 (-19.0, -0.80) | -0.1 (-0.59, 0.39) |
| GMV | -5.5 (-12.47, -1.47) | -0.3 (-1.00, 0.43) |
| WMV | -4.4 (-9.82, 1.08) | 0.1 (-0.42, 0.70) |
| HV | -0.2 (-0.35, -0.03) | -0.2 (-0.86, 0.49) |
| WMFA | -0.2 (-0.62, 0.25) | -0.2 (-1.39, 0.96) |
| **ST (highest vs. lowest quartile) → logBDNF** | 0.0 (-0.21, 0.31) | N/A |
| **logBDNF → MRI** |  |  |
| TBV | -0.7 (-3.71, 2.30) | 0.3 (0.16, 0.47) |
| GMV | -0.6 (-2.91, 1.70) | 0.4 (0.13, 0.58) |
| WMV | -0.1 (-1.90, 1.70) | 0.2 (0.07, 0.43) |
| HV | 0.0 (-0.03, 0.08) | 0.5 (0.28, 0.71) |
| WMFA | -0.1 (-0.20, 0.09) | -0.6 (-0.92, -0.19) |
| **ST (highest vs. lowest quartile) → logBDNF → MRI** |  |  |
| TBV | -9.8 (-18.93, -0.76) | -0.1 (-0.60, 0.36) |
| GMV | -5.5 (-12.45, -1.50) | -0.3 (-1.00, 0.40) |
| WMV | -4.4 (-9.83, 1.1) | 0.1 (-0.44, 0.68) |
| HV | -0.2 (-0.35, -0.03) | -0.2 (-0.88, 0.43) |
| WMFA | -0.2 (-0.62, 0.26) | -0.1 (-1.31, 1.01) |
| **Hypothesis 2: Moderation** |  |  |
| **ST (highest vs. lowest quartile) and logBDNF moderation** |  |  |
| TBV | 8.5 (-0.13, 17.11) | -0.2 (-0.60, 0.30) |
| GMV | 2.2 (-4.48, 8.83) | -0.2 (-0.81, 0.50) |
| WMV | 6.3 (1.14, 11.49) | -0.2 (-0.68, 0.37) |
| HV | 0.1 (-0.10, 0.21) | -0.3 (-0.87, 0.37) |
| WMFA | 0.3 (-0.11, 0.72) | 0.2 (-0.86, 1.30) |

Note.

1. BDNF=brain-derived neurotrophic factor, ST=sedentary time, TBV=total brain volume, GMV=grey matter volume, WMV=white matter volume, HV=hippocampal volume, WMFA=white matter fractional anisotropy.
2. Results of Model 2 (additionally adjusted for vascular risk factors and education) were similar as Model 1, except that: Significant moderation effects were only found for WMV. For the main analysis, β coefficients were for ST on logBDNF, MRI (cross-sectional) or change of MRI (longitudinal). For the moderation models, β coefficients were for interaction. For the mediation analysis, β coefficients were partially standardized indirect effects.

**Table 2_Supp. Comparison of missingness in characteristics of participants at the Year 25, CARDIA Brain-MRI sub-study**

| **Characteristics** | **Year 30**  **(n=418)** | **Missing in Year 30**  **(n=194)** |
| --- | --- | --- |
| **Age, mean(SD), y** | 50.5±3.4 | 50.0±3.7 |
| **Female No.(%)** | 216 (51.7) | 100 (51.6) |
| **Black No.(%)** | 153 (36.6) | 83 (42.8) |
| **Education No.(%)** |  |  |
| High school | 81 (19.5) | 50 (25.8) |
| College | 243 (58.4) | 113 (58.3) |
| Graduate | 92 (22.1) | 31 (16.0) |
| ***BMI, mean(SD), kg/m^2^** | 27.6±4.7 | 31.1±6.9 |
| ***Diabetes No.(%)** | 34 (8.1) | 194 (17.0) |
| ***Dyslipidemia No.(%)** | 150 (35.9) | 85 (44.0) |
| ***Hypertension No.(%)** | 119 (28.5) | 79 (40.7) |
| ***Smoking No.(%)** | 147 (35.5) | 93 (48.4) |
| **Depression score, mean(SD)** | 8.3±6.8 | 9.3±7.3 |
| **BDNF plasma, mean(SD), pg/ml** | 2973.0±2862.7 | 3342.1±3401.2 |
| ***Sedentary time (hours/day), No.(%)** | | |
| ≤4.3 | 111 (26.6) | 41 (21.1) |
| 4.3 - 5.9 | 110 (26.3) | 44 (22.7) |
| 5.9 - 8.4 | 106 (25.4) | 46 (23.7) |
| >8.4 | 91 (21.8) | 63 (32.5) |
| **Brain measures, % of ICV(SD)** | | |
| TBV | 85.0±2.7 | 85.2±3.1 |
| GMV | 46.8±0.2 | 46.7±2.4 |
| WMV | 38.3±1.5 | 38.5±1.7 |
| HV | 0.6±0.0 | 0.6±0.1 |
| WMFA, % (SD) | 31.2±1.8 | 31.0±1.9 |

Note.

1. BDNF=brain-derived neurotrophic factor, BMI=body mass index, ICV=intracranial volume, TBV=total brain volume, GMV=grey matter volume, WMV=white matter volume, HV=hippocampal volume, WMFA=white matter fractional anisotropy. Missingness: Education n=2; Dyslipidemia n=1; Smoking n=6; Depression score n=3.

*p<0.05, adjusted for age, sex, and race.

**Table 3_Supp. Associations between sedentary time and 25-year exam risk factors, CARDIA Brain-MRI sub-study**

| **Risk factor** | **β or Odds Ratio estimate** | **95% CI** |
| --- | --- | --- |
| **BMI** | 3.01 | 1.73, 2.55 |
| **Diabetes** | 3.39 | 1.33, 8.33 |
| **Smoking** | 2.05 | 1.26, 3.35 |
| **Hypertension** | 2.91 | 1.70, 4.99 |
| **Dyslipidemia** | 2.05 | 1.24, 3.38 |
| **Depression** | 1.56 | -0.09, 3.21 |

Note.

1. BDNF=brain-derived neurotrophic factor, BMI=body mass index.
2. β estimate was the difference of one-unit change in BMI or depression score for highest vs. lowest sedentary time quartiles and Odds Ratio estimate was the risk of the presence of diabetes, smoking, hypertension, and dyslipidemia, adjusted for age, race, and sex.
