# Supplementary figures and images for: Sedentary behavior, brain-derived neurotrophic factor and brain structure in midlife: A longitudinal brain MRI sub-study of the coronary artery risk development in young adults study
Source: Front Dement. 2023 Mar 13;2:1110553. doi: 10.3389/frdem.2023.1110553 (PMC11285629; doi:10.3389/frdem.2023.1110553)

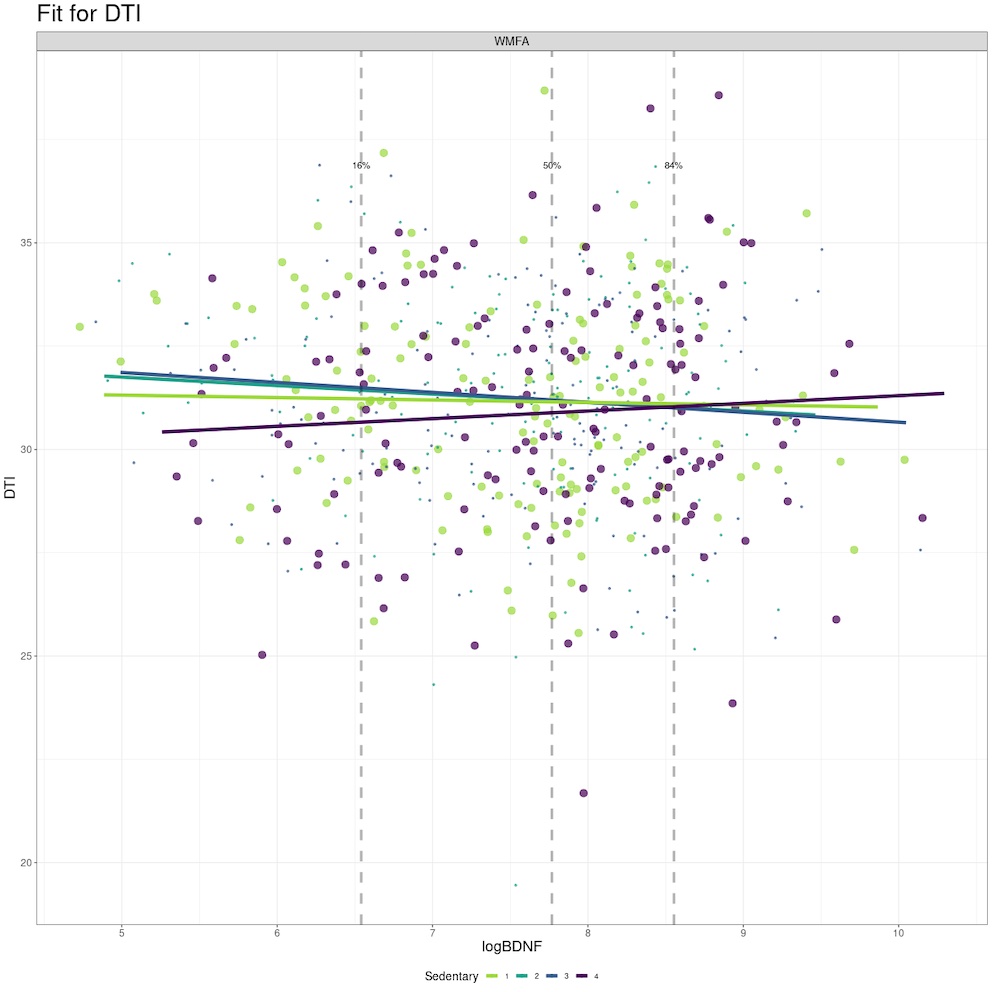

Supplement: Supplementary file 2 [file Image_1.JPEG]

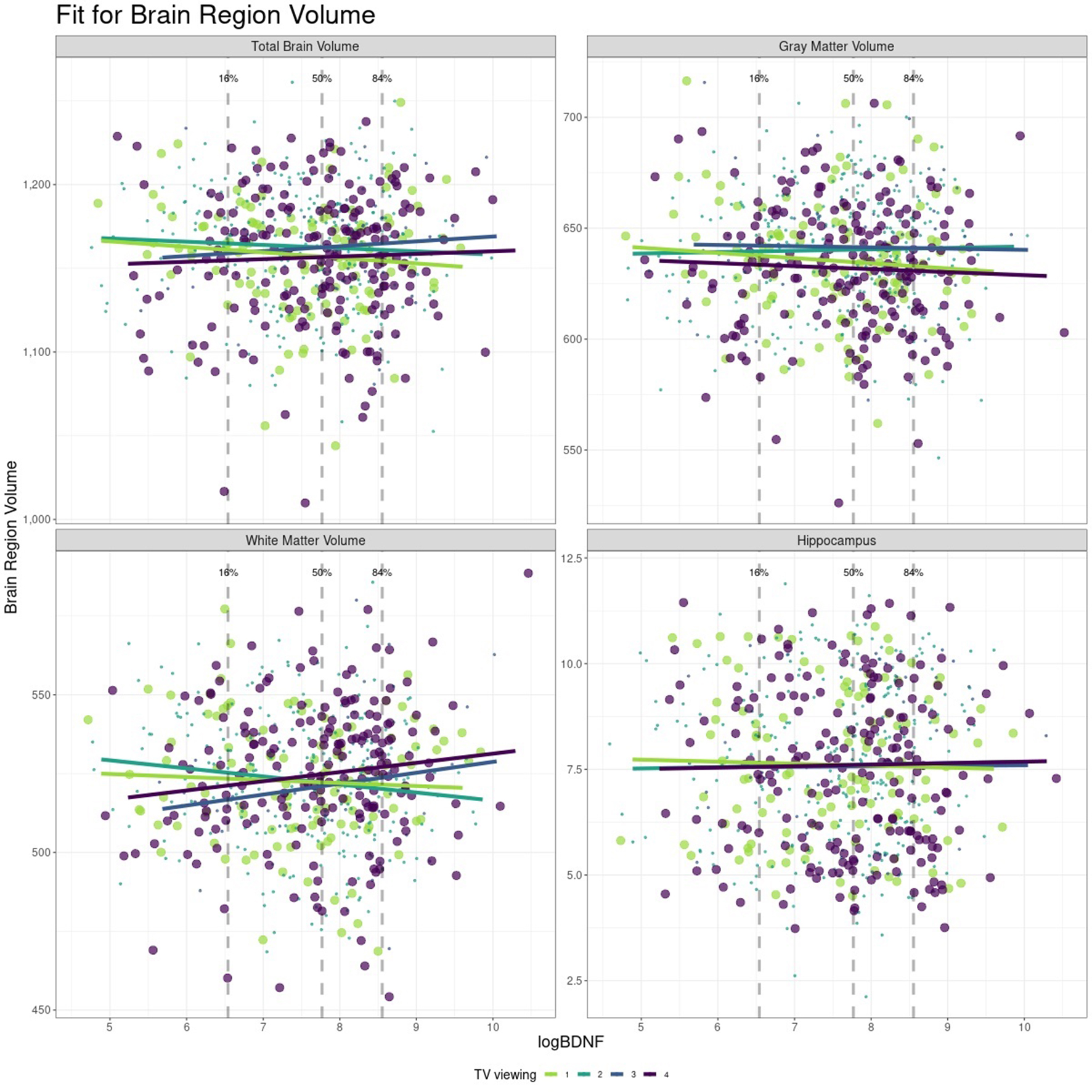

Supplement: Supplementary file 3 [file Image_2.JPEG]
